# Utilization of single nucleotide polymorphism array in prognostication of cutaneous melanocytic tumor with CRTC1::TRIM11 fusion

**DOI:** 10.1016/j.jdcr.2025.07.002

**Published:** 2025-07-18

**Authors:** Stephanie Humen, Aziz Khan, Gillian Weston

**Affiliations:** a University of Connecticut School of Medicine, Farmington, Connecticut; b Department of Dermatology, University of Connecticut, Farmington, Connecticut

**Keywords:** clear cell sarcoma, cutaneous melanocytic tumor with CRTC1::TRIM11 mutation, melanoma, SNP array

## Introduction

Cutaneous melanocytic tumor with *CRTC1::TRIM11* fusion (CMTCT), first reported by Cellier et al in 2018,^1^ is an intradermal, nonpigmented tumor with melanocytic differentiation and characteristic *CRTC1::TRIM11* fusion. Since its discovery, a total of 49 cases have been reported,^2^ most demonstrating indolent behavior.^1^^,^^2^ However, 2 reports of metastatic disease and 1 local recurrence have been recorded.^1^^,^^2^ Given the potential for metastasis, complete excision may be good practice, although standard management guidelines have not been established. Additional molecular studies may aid in prognostication. Here, we present a case of digital CMTCT in which single nucleotide polymorphism (SNP) array analysis and telomerase reverse transcriptase gene (TERT) mutation analysis guided clinical decision-making.

## Case report

**Fig 1:**
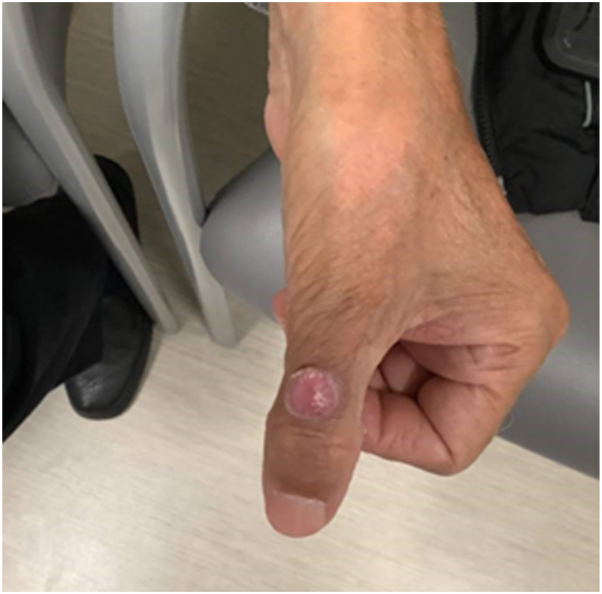
Clinical image of 72-year-old male patient's solitary pink to red, soft, round nodule with collarette scale on the left dorsal thumb.

**Fig 2:**
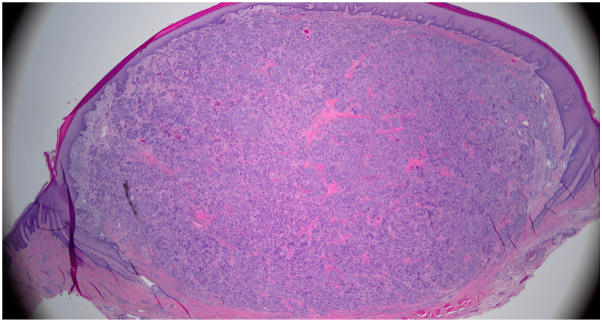
Histopathologic features of cutaneous melanocytic tumor with *CRTC1::TRIM11* fusion illustrating a well-circumscribed dermal proliferation of epithelioid and spindle cells with eosinophilic cytoplasm and vesicular nuclei prepared with hematoxylin-eosin stain. Original magnification 100×.

A 72-year-old South Asian male with a medical history of bladder cancer, stage 3 chronic kidney disease, hepatitis B, and hypertension presented with a 1.5-year history of a painless growth on the left dorsal thumb. Physical examination revealed a 1 cm × 1 cm pink to red, round, soft nodule with a well-delineated collarette scale (Fig 1). Histopathological examination of a shave biopsy showed a well-circumscribed nodule within the dermis composed of intersecting aggregates and fascicles of spindle-shaped and epithelioid cells set within a fibrotic stroma (Fig 2). Some fascicles displayed herringbone-like palisading nuclei. Tumor cells displayed enlarged vesicular nuclei with prominent nucleoli and abundant staining vacuolated cytoplasm. No significant pleomorphism or mitoses were identified. The epidermis was not involved. Immunohistochemical staining was diffusely positive for Sox10, MITF, and epithelial membrane antigen. There was patchy positivity for S100 and MART1. The cells were negative for p63, AE1/AE3, CAM5.2, smooth muscle actin, desmin, human melanoma black-45, and preferentially expressed antigen in melanoma. PPH3 revealed a low proliferative index. A diagnosis of clear cell sarcoma was considered; however, fluorescence in situ hybridization analysis did not detect the characteristic Ewings sarcoma gene rearrangement seen in this entity. A sarcoma next-generation sequencing panel (Cleveland Clinic) demonstrated characteristic *CRTC1::TRIM11* fusion.

The tumor did not extend to the biopsy edges examined; however, the margins were narrow. The location of the tumor on the dorsal aspect of the thumb made excision potentially problematic. To better estimate the tumor's biologic potential, additional molecular diagnostic testing was performed. SNP array (Chromosomal Microarray Analysis-Melanoma, Affymetrix OncoScan FFPE, Michigan Medicine Pathology and Clinical Laboratories) revealed 5 copy number variations (CNVs), including a 1.8 Mb loss on chromosome 1q42.13 spanning TRIM11 gene rearrangement detected in prior genetic analysis. SNP array helps to classify and prognosticate histologically ambiguous melanocytic neoplasms, with malignant neoplasms typically showing a higher number of CNVs. One study had an average of 18 CNVs in melanomas, with benign nevi in the same study with 0 CNVs.^3^ While some studies propose an upper limit cut-off of 3-4 CNV to distinguish benign from malignant melanocytic neoplasms, others suggest 6 CNV in certain melanocytic proliferations.^3^, ^4^, ^5^ These algorithmic approaches may not apply to tumors with partial melanocytic expression like CMTCT. Our case demonstrated 5 CNVs, which were, in the context of the detected gene rearrangement (ie, not a conventional melanocytic tumor), considered a borderline abnormality. Additional genomic testing for TERT mutation was undertaken and was negative.

In summary, the histomorphology, identified gene rearrangement, and negative TERT mutation status did not support high malignant potential. As observed, tissue margins were free of tumor, and there was no definitive evidence in support of aggressive tumor behavior; the patient chose to defer surgical intervention. He is without clinical recurrence during the 30-month follow-up period since initial shave removal.

## Discussion

CMTCT is a novel neoplasm that often presents nonspecifically as a skin-colored lesion occurring on the extremities. Histologically, it is characterized by a predominantly dermal neoplasm comprised of fascicles of epithelioid to spindle-shaped cells. Immunohistochemical staining often demonstrates a melanocytic profile.^1^^,^^6^ These findings raise the differential diagnosis of metastatic melanoma and clear cell sarcoma. While the lack of epidermal involvement and absence of significant pleomorphism help differentiate histological features, molecular sequencing modalities targeted at CRTC1 and TRIM11 are essential in discriminating CMTCT from these other entities.^3^^,^^6^^,^^7^ CRTC1 is a transcription factor acting as a cyclic adenosine monophosphate response element-binding protein to transcribe target genes involved in the cell cycle, while TRIM11 is an E3 ubiquitin ligase responsible for targeting misfolded proteins.^1^^,^^7^ The function of the fusion protein, *CRTC1::TRIM11* is widely unknown. One proposed mechanism is that the site of interaction between the 2 proteins deletes the site of TRIM11 responsible for protein degradation, promoting tumorigenesis.^2^^,^^4^

Further, molecular diagnostic testing is useful beyond identification of the characteristic fusion. SNP arrays detect CNVs and allelic homozygosity/loss of heterozygosity and are useful in characterizing the biologic potential of a wide variety of tumors. Across melanocytic tumors and other tumor types in the field of pathology, there is a direct correlation between CNV burden and tumor behavior, such that increased CNV is associated with greater metastatic potential, while limited CNV tends to reflect an indolent course. While various studies have aimed to develop algorithmic, objective approaches to interpretation of CNV number in certain diagnostic dilemmas, additional study is needed to understand CNV results across a span of tumors and will be helpful to fully characterize and prognosticate tumors, including and especially those, like CMTCT, which are relatively newly described. Additionally, mutations in TERT promoter genes occur frequently in a variety of malignancies, including melanomas, and when detected, suggest the capability of infinite cell growth and therefore more aggressive behavior.^8^

While most of the 49 reported cases of CMTCT behaved indolently, there are few reports of local recurrence and lymph node metastasis^1^; no cases reported use of SNP array. In our case, an intermediate number (5) of CNVs detected on the SNP array and the absence of a TERT mutation were reassuring and helped guide clinical decision-making. Without any intervention beyond initial shave removal, our patient has been without recurrence after 20 months of follow-up; conservative management, supported by molecular diagnostics, seems to have been appropriate in this case.

As more cases are diagnosed and reported, molecular diagnostics, beyond those that simply identify characteristic fusions/mutations, may help better characterize the biological potential of this relatively newly described entity and inform better prognostication and management strategies.

## Conflicts of interest

None disclosed.
